# HIV policy implementation challenges: Experiences of HIV managers in primary healthcare facilities in the Western Cape

**DOI:** 10.4102/curationis.v49i1.2787

**Published:** 2026-03-12

**Authors:** Furaha Akimanimpaye, Talitha Crowley, Verinia Titus

**Affiliations:** 1 School of Nursing, Faculty of Community and Health Sciences, University of the Western Cape, Cape Town, South Africa; 2 Western Cape College of Nursing, Cape Town, South Africa

**Keywords:** HIV policy implementation, primary healthcare, health information systems, health systems, human resources, infrastructure, training, South Africa

## Abstract

**Background:**

Implementing HIV policy involves translating policy goals into practical action. In the Western Cape, South Africa, the lack of clear guidelines has challenged the consistent implementation of HIV policies at primary healthcare (PHC) facilities.

**Objectives:**

This study aimed to explore HIV managers’ experiences regarding the challenges of HIV policy implementation at PHC facilities within the Northern Tygerberg sub-district, Cape Metropole, Western Cape.

**Method:**

An exploratory, descriptive, qualitative design was employed. Purposive sampling was applied to select HIV managers (*n* = 10) with experience in policy implementation. Data were collected through semi-structured interviews and analysed using thematic analysis.

**Results:**

Four themes emerged from the study, reflecting the challenges experienced by HIV managers during the implementation of HIV policies at PHC facilities, including human resource challenges, staff knowledge and attitudes, system readiness, and training.

**Conclusion:**

Effective implementation of HIV policy remains essential to the national HIV response. While significant targets have been achieved, ongoing systemic challenges continue to hinder optimal policy execution and the delivery of services.

**Contribution:**

The study highlights the critical need for stronger human resource systems, improved training platforms, and enhanced facility infrastructure to ensure effective HIV policy implementation and better health outcomes for individuals living with HIV.

## Introduction

Human Immunodeficiency Virus (HIV) continues to pose a significant global public health challenge. As of 2023, an estimated 39.9 million people were living with HIV worldwide, and approximately 630 000 lives are lost annually to acquired immunodeficiency syndrome (AIDS)-related complications, making it one of the leading causes of mortality (UNAIDS 2025). Sub-Saharan Africa bears the greatest burden, with South Africa alone accounting for approximately 8 million people living with HIV (Statistics South Africa 2024). The impact of HIV extends beyond health, deeply affecting the socio-economic development of individuals, families and communities, particularly in sub-Saharan Africa, where it is a leading contributor to adult mortality (Ward et al. 2019). Although global efforts to curb the epidemic have led to substantial progress, including the wide-scale rollout of antiretroviral therapy (ART), HIV remains a persistent challenge and is likely to remain so for years to come (Ford et al. 2018). While ART has transformed HIV into a manageable chronic condition, a definitive cure or vaccine is still unavailable (Kharsany & Karim 2016).

The policy context guiding HIV service delivery in South Africa is informed by several key national frameworks. These include the National Strategic Plan for HIV, tuberculosis (TB) and sexually transmitted infections (STIs) 2023–2028 (South African National AIDS Council 2023), the Integrated Clinical Services Management (ICDM) framework (National Department of Health 2016a), and the most recent National HIV Programme Guidelines (National Department of Health 2023). Together, these documents set out the standards for HIV prevention, testing, treatment initiation, chronic care integration, and adherence support within the primary healthcare (PHC) platform. They also define the operational expectations for PHC facilities and managers, ensuring alignment with national priorities, integrated chronic care processes and updated clinical practice. In addition, the Department of Health’s Knowledge Hub provides training resources and implementation tools that support health workers in applying these policies in practice (National Department of Health 2016a). These frameworks collectively provide the policy and operational foundations within which PHC facilities are expected to implement HIV services.

In response to the ongoing burden, the World Health Organization (WHO) introduced the 95-95-95 targets, which aim to ensure that 95% of all people living with HIV know their status, 95% of those diagnosed receive sustained ART, and 95% of those on treatment achieve viral suppression. The Universal Test and Treat (UTT) strategy was introduced to support this goal, recommending immediate initiation of ART for all HIV-positive individuals, irrespective of their CD4 count (United Nations Programme on HIV/AIDS 2021; WHO 2016). South Africa, which hosts the world’s largest ART programme, implemented decentralised HIV care through PHC facilities to widen access to treatment and improve service delivery (South African History Online 2019). This model assigns responsibility to healthcare professionals, including medical officers, nurses and pharmacists, to initiate treatment for eligible patients at the PHC level while referring complex cases to higher levels of care (Makhado, Davhana-Maselesele & Ramukumba 2020).

Given the evolving nature of HIV treatment, healthcare workers are expected to stay informed about policy updates, revised treatment guidelines and the management of comorbidities such as tuberculosis. The effective implementation of these policies is essential for improving HIV care quality, reducing new infections and lowering HIV-related morbidity and mortality (National Department of Health 2016b). Despite the regular revision of HIV policies and guidelines, evidence suggests gaps in their implementation, which undermines the success of HIV programmes (Wang et al. 2019).

It has been suggested that ineffective policy implementation may result from inadequate planning and weak quality assurance systems (Ajulor 2018). In addition, poor communication regarding policy updates to key stakeholders, including HIV managers, healthcare workers and non-governmental organisations (NGOS), can lead to inconsistent application of policies across facilities (Shariff 2014). Recent studies in LMICs confirm that weak institutional support, limited capacity building and fragmented feedback systems continue to undermine effective HIV policy implementation (Mishra et al. 2023; Simooya et al. 2023; Upadhyay et al. 2023). However, despite these insights, there is limited empirical evidence on how HIV managers in South Africa specifically experience and navigate such barriers within PHC settings. Given the country’s high HIV burden and its ongoing push to strengthen the translation of policy into practice, understanding these managerial perspectives remains crucial.

### Problem statement

In the Northern Tygerberg sub-district, HIV managers are working under growing pressure as programme performance remains below expectations, with retention in care dropping from 71% at initiation to 49% within the first few years (Western Cape Government 2018). Many of these managers describe working in settings where staff shortages, limited space and competing service demands make it difficult to bring national HIV policies to life at the facility level. Their experiences echo findings from across South Africa and other low- and middle-income countries, where weak institutional support, limited training opportunities and fragmented feedback systems repeatedly hinder effective policy implementation (Mishra et al. 2023; Sheikh, Abimbola & Hill 2020; Simooya et al. 2023). National evidence similarly points to ongoing gaps in retention, linkage to care and guideline adherence within PHC services (HSRC 2022; Modipane, Lebese & Maputle 2023; Nicol, Bimerew & Allie 2023). When new or updated HIV guidelines reach facilities without clear guidance, mentorship or sustained support from district HIV, AIDS, STIs and Tuberculosis (HAST) teams, managers are often left to interpret and implement policies on their own (Farokhzadian, Nayeri & Borhani 2018; Lujintanon, Chawla & Vella 2024). Despite this, little is known about how HIV managers in the Northern Tygerberg sub-district actually make sense of these policies and navigate these daily challenges. Understanding their perspectives is essential for improving how HIV policies are supported and implemented in the Western Cape.

### Research purpose

This study aimed to explore the experiences of HIV managers regarding the challenges of implementing HIV policies at PHC facilities in the Northern Tygerberg sub-district.

### Significance of the study

The findings of this study may contribute to improving the implementation of HIV policy by providing context-specific recommendations tailored to the PHC facility level. In addition, the insights gained hold relevance for nursing education, as they can inform the development and enhancement of undergraduate and postgraduate curricula, particularly in areas related to HIV policy implementation. Furthermore, these findings can serve as a valuable foundation for future research in this field.

## Research methods and design

This study used a qualitative exploratory–descriptive design to gain insight into how HIV managers navigate the day-to-day realities of implementing HIV policies in PHC facilities. This approach was chosen because it allows researchers to explore real-life experiences without forcing participants’ accounts into a strict theoretical framework (Colorafi & Evans 2016; Hunter, McCallum & Howes 2019). It also enabled participants to express their views in their own words, with an inductive analytic process helping the research team interpret these experiences as they emerged (Braun & Clarke 2019).

Structured models such as the updated Consolidated Framework for Implementation Research (CFIR 2.0) can offer valuable guidance for examining multilevel implementation issues across a health system (Damschroder et al. 2022). However, this study was intentionally designed to focus on operational-level experiences, and therefore, CFIR was not adopted as the main framework. Its usefulness for future studies exploring broader, system-wide implementation dynamics is acknowledged as a limitation.

### Setting

This study was conducted in the Northern-Tygerberg substructure of the Western Cape province, South Africa, a metropolitan area comprising 144 Department of Health and 4 City of Cape Town PHC facilities. The study sites were selected purposively at the sub-district level based on documented gaps in HIV policy implementation identified in routine audit reports. Thus, data collection sites were Community Day Clinics operating on an 8-h daily schedule. Each facility had its own HIV Operational Manager functioning within the broader PHC structure.

### Population and sampling

This study focused on middle-management HIV programme staff in the Department of Health’s PHC system, including Operational Managers (PHC), Sub-district HAST Programme Coordinators and Sub-district Clinical Governance Managers. These managers were well positioned to share practical insights because they work between frontline clinicians and sub-district leadership, where they translate HIV policies into daily practice and oversee programme performance. To keep the focus on operational-level experiences, frontline clinicians and senior provincial or district managers were not included. Participants were eligible if they worked in the Northern Tygerberg sub-district, were directly involved in HIV policy implementation and monthly audit verification, and had at least 2 years of experience in an HIV management or oversight role (Bhardwaj 2019; Burns, Gray & Grove 2018). Managers with less than 2 years’ experience or without responsibility for HIV audit processes were excluded. Using a purposive, homogeneous sampling approach, twelve managers who met these criteria and had first-hand knowledge of programme implementation were selected (Saunders, Lewis & Thornhill 2012).

### Pilot study

Two pilot interviews were conducted to assess the suitability and clarity of the interview guide. These helped to refine the wording and the flow of the questions and ensured that interviews’ length was appropriate. The development of the interview guide was informed by the study objectives, an exploratory review of literature on HIV policy implementation, and qualitative interview principles (Creswell & Creswell 2018; Kallio et al. 2016). The guide was designed to elicit managers’ perspectives on their roles, challenges and experiences related to implementing HIV policies. Two pilot interviews were conducted in May 2022 and ensured that the questions aligned with the research focus, allowed for effective probing, and were appropriate for addressing the aim of the study (Ismail, Kinchin & Edward 2017). The pilot interviews were not included in the final analysis, and the guide was refined to improve question flow and coherence.

### Data collection

Participants were recruited with the assistance of sub-district PHC and facility managers, who acted as gatekeepers by identifying eligible HIV managers. V.T. then conducted face-to-face semi-structured interviews with managers working in PHC facilities in the Northern Tygerberg sub-district. Gatekeepers introduced the study, and the researcher followed up via email to provide study details and arrange the interviews. Of the 12 managers approached, 10 took part; 1 declined, and 1 was excluded because their facility only offered HIV testing. Interviews were conducted in English, in private rooms, between May and August 2022. Only aggregated demographic information was collected, and no identifying details were recorded, ensuring full anonymity. The semi-structured format supported open, in-depth discussion (Brink, Van der Walt & Van Rensburg 2018; Creswell & Creswell 2018), beginning with the question: ‘Can you describe your general experience with the policy implementation process in your context?’. Interviews lasted 45–60 min, were scheduled at times convenient for participants, and were audio-recorded with informed consent. Data saturation was reached after eight interviews, and two additional interviews confirmed this, resulting in a final sample of ten participants.

### Data analysis

Audio recordings were transcribed verbatim and analysed using inductive thematic analysis guided by Braun and Clarke’s six steps. Firstly, the researchers familiarised themselves with the data through repeated reading of the transcripts and initial note-taking to gain a holistic understanding of participants’ perspectives. Secondly, initial codes were generated to capture meaningful and recurring features of the data relevant to the research aims. Thirdly, these codes were systematically applied across the entire dataset to ensure consistent and comprehensive coding. Fourthly, coded segments were collated and examined to identify potential themes, allowing the researchers to explore patterns and relationships within the data. Fifthly, themes were refined, defined, and named, with attention to ensuring conceptual clarity and distinctiveness between themes. Finally, the data were interpreted by situating the themes within the broader context of the study, enabling the development of a coherent and insightful narrative that captured the essence of the phenomenon under investigation.

### Trustworthiness

To ensure the trustworthiness of this study, Lincoln and Guba’s (1965) criteria, credibility, dependability, confirmability and transferability, were carefully applied. Credibility was strengthened through member checking, where participants were invited to review the researcher’s summaries and clarify or expand on their responses. Dependability was supported by keeping a clear audit trail, including coding records and checked transcripts. Confirmability was enhanced through reflexive practices such as team discussions, which helped to minimise researcher bias and ensure that the findings reflected participants’ views rather than researcher assumptions; any differences in coding or interpretation were resolved through consensus. Transferability was addressed by providing a rich description of the study context and participants’ characteristics, along with verbatim quotations, enabling readers to determine the relevance of the findings to their own settings. Data collection and coding were conducted by V.T. under the supervision of T.C. and F.A., who verified the coding, sub-themes and themes to maintain consistency. Any discrepancies were discussed and resolved, ensuring an unbiased and credible interpretation of the data.

### Ethical considerations

This study received ethical approval from the University of the Western Cape’s Biomedical Research and Ethics Committee (reference number: BM20/10/6) and the Western Cape Department of Health (reference number: WC202112_013) and was conducted in accordance with the Department of Health’s research ethics guidelines. The study adhered to the core ethical principles of autonomy, beneficence, non-maleficence and justice. Autonomy was upheld through a clear and thorough informed consent process: participants were fully briefed on the purpose of the study, the voluntary nature of their involvement, and their right to withdraw at any stage without consequence. Privacy and confidentiality, central to the principles of non-maleficence and respect for persons, were ensured by anonymising all data, removing identifying information from transcripts, and securely storing audio recordings and research documents. Beneficence was promoted by designing the study to generate meaningful insights while minimising any burden placed on the participants. Non-maleficence was further ensured by preventing harm, including emotional or psychological discomfort, through sensitive interviewing practices and careful handling of personal information. Justice was upheld through fair recruitment procedures, ensuring that all eligible participants had an equitable opportunity to take part in the study.

## Results

### Participant characteristics

A total of 10 HIV managers participated in the study. The majority of participants were female (*n* = 9), with only one male participant. The years of experience in HIV management roles varied, ranging from 2 years to 17 years. Most participants had extensive experience, with eight of the ten having more than 6 years of service in their positions, the range being 2–17 years.

## Themes

**FIGURE 1:**
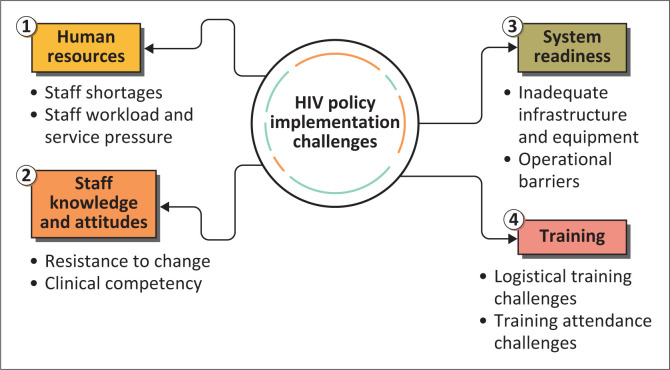
Themes and sub-themes.

Through the analysis of the data, four themes were identified, including: (1) Human Resource Challenges, (2) Staff Knowledge and Attitudes, (3) System Readiness and (4) Training (Figure 1).

### Theme 1: Human resources challenges

Human resources emerged as a critical factor influencing the successful implementation of HIV policies within the PHC setting. The availability of skilled staff and adequate human resource capacity is essential in ensuring that service delivery meets both clinical standards and policy targets. However, this study highlighted persistent staffing shortages and workload pressures that hinder effective implementation.

#### Sub-theme 1.1 Staff shortages

Participants consistently identified staff shortages as a recurring barrier to policy implementation. Routine staff absences because of annual leave, sick leave or unplanned absenteeism often left facilities operating with minimal personnel. This situation placed significant strain on the quality of care and jeopardised the achievement of set targets, particularly in areas with growing patient numbers.

One participant explained the impact of staff shortages on daily operations:

‘We struggle with staff, because sometimes the amount of staff allocated to the ART [*antiretroviral therapy*] area … It’s just enough to get through the day. So, if it is that there are new things, you might need more staff, so staff, in general, becomes a challenge.’ (Participant 3, female, 10 years’ experience)

The increasing patient load further compounded this challenge, forcing healthcare teams to rotate staff in an attempt to balance service coverage. This often meant that clients without appointments could not be accommodated and service targets occasionally remained unmet:

‘As you have your day-to-day, you have your service pressures, there is a continuous increase [*in the*] flow of things … Staff structures that have been adjusted.’ (Participant 7, female, 9 years’ experience)

#### Sub-theme 1.2 staff workload and service pressure

In addition to staffing shortages, participants highlighted the growing workload as a significant concern. New policy implementation often required additional administrative and clinical responsibilities, extending the time staff spent with each patient and reducing the total number of clients seen per day. As a result, waiting times increased, and staff were frequently unable to meet daily targets. One HIV manager described how these time pressures affected both staff performance and patients’ experience:

‘Time constraints sometimes are against us. If staff have implemented this new policy, it sometimes takes a little bit longer for them to spend … they spend a bit more time with a client, resulting in extended waiting time for the patients and their workload is also influenced because they often can see a little bit less.’ (Participant 6, female, 12 years’ experience)

Another participant shared how the limited number of staff forced healthcare workers to assume multiple roles during the same shift:

‘Hundreds of patients were coming through the gate before we could even see them, and we had to explain the situation to the patients. The same sister who was doing the screening was also the one seeing them inside. So yes, there wasn’t anyone else; it was the same staff responsible for PREP [*pre-exposure prophylaxis*] as well.’ (Participant 7, female, 9 years’ experience)

Some HIV managers actively supported their teams by stepping into clinical roles themselves. This hands-on approach not only reduced the immediate patient backlog but also allowed managers to understand the challenges faced by frontline staff:

‘How can I assist? Because if it’s something that I need to work through with them for a while, I want to see firsthand whether there are any challenges. I won’t just hand it over to them; I will physically work alongside them to understand the challenges and see how I can assist.’ (Participant 3, female, 10 years’ experience)

### Theme 2: Staff knowledge and attitudes

The knowledge and attitudes of healthcare staff are pivotal in determining the success of policy implementation. Staff who possess strong clinical skills and experience tend to adapt more readily to new policy directives, especially when they are supported by targeted training opportunities. However, the attitude of staff towards the process plays an equally significant role. While a positive attitude can foster resilience and adaptability during transitions, negative attitudes often lead to reluctance, reduced engagement, and ultimately poor implementation outcomes.

#### Sub-theme 2.1 resistance to change

One of the prominent barriers to policy implementation highlighted by participants was staff resistance to change. Several participants explained that unfamiliarity with new practices, uncertainty about expected outcomes and limited exposure to new interventions often contributed to reluctance. Staff members were particularly hesitant to adopt changes when they were unsure about the safety, effectiveness or sustainability of a new approach, such as introducing unfamiliar ART regimens or new clinical protocols.

As one participant observed:

‘When something is new, people often hesitate because they are unsure whether it will succeed. Many staff prefer to wait until they’ve seen evidence of success in another facility before committing to the change themselves. Few want to be the first to take the risk.’ (Participant 7, female, 9 years’ experience)

The findings suggest that leadership engagement and open communication are essential in addressing these fears. Participants emphasised the importance of creating safe spaces for staff to voice their concerns, and for managers to provide reassurance and clarity regarding policy changes. One participant highlighted this by stating:

‘It largely comes down to leadership. When leaders actively share information, listen to staff concerns, and acknowledge their fears, the transition becomes smoother and staff feel more secure in adopting new practices.’ (Participant 7, female, 9 years’ experience)

#### Sub-theme 2.2 clinical competency

Clinical competency emerged as another critical factor influencing policy implementation. Competency ensures the delivery of quality care, promotes sound clinical decision-making, and contributes to patient safety and satisfaction. However, gaps in staff experience or insufficient training can result in uncertainty and hesitation, particularly when introducing new services or clinical procedures.

A manager explained the importance of assessing staff preparedness before assigning new responsibilities:

‘You need to assess whether staff have the right skills and training before asking them to implement a new policy. As a manager, it’s your responsibility to understand their level of competence and guide them accordingly.’ (Participant 7, female, 9 years’ experience)

In addition, several participants highlighted that fear of adverse patient outcomes, such as immune reconstitution inflammatory syndrome (IRIS) following ART initiation, often amplified staff anxiety and delayed confidence in clinical practice. Ongoing mentorship and direct exposure to ART management were identified as key to overcoming these fears.

As one participant reflected:

‘What I noticed was that the staff’s hesitation was more fear-based, especially around starting ART [*antiretroviral therapy*] in patients who might develop IRIS [*immune reconstitution inflammatory syndrome*]. Once they gained more experience and had support through the process, their confidence improved, and the fear diminished.’ (Participant 1, female, 11 years’ experience)

### Theme 3: System readiness

The effective implementation of health policies depends not only on human resources but also on the availability of essential infrastructure, functional equipment, adequate stationery and reliable health information systems. In this study, participants highlighted several system barriers that compromised the ability of healthcare facilities to implement new policies efficiently.

#### Sub-theme 3.1: Inadequate infrastructure and equipment

One of the most prominent challenges reported by participants was the physical limitation of healthcare facility infrastructure. Many facilities lacked sufficient space to accommodate growing patient numbers, which often forced staff to manage clinical services in makeshift spaces such as prefabricated buildings. The inability to expand existing infrastructure placed immense strain on staff and undermined service delivery.

A participant illustrated this challenge by stating:

‘Now, as our clientele increases daily, we don’t have enough space to put the extra clinician in the area. So, they become overloaded and overwhelmed with the number of patients coming to that specific area. So, infrastructure is a major challenge.’ (Participant 3, female, 10 years’ experience)

Beyond physical space, issues with digital infrastructure and information systems were commonly reported. Participants described frequent problems with outdated or incompatible IT hardware and software, poor network stability, and an overall lack of computer literacy among staff, all of which slowed service delivery and interfered with the efficient implementation of policies:

‘With everything being computerised, you often are stuck with network problems or software problems, or you often can get a staff member who is not computer literate. So, then you must make sure that the staff are trained, and that can also slow down your progress of implementing a policy.’ (Participant 4, female, 17 years’ experience)

Furthermore, participants raised concerns about supply chain inefficiencies, which hindered the timely availability of essential medical supplies, equipment and stationery. Proper coordination between support services and clinical teams was identified as crucial to ensuring a smooth policy rollout:

‘All supporting departments need to be aligned, and the supply chain system must be responsive enough to meet the demands that come with new policy implementation.’ (Participant 7, female, 9 years’ experience)

#### Sub-theme 3.2: Operational barriers

Operational issues further compounded the challenges faced during policy implementation. Participants specifically mentioned the negative impact of frequent power outages, including loadshedding, on both service continuity and electronic systems. Even after power was restored, network disruptions often persisted, delaying access to digital records and hampering clinical workflows:

‘Power cuts, especially loadshedding, you have a power cut, and we know load shedding is one of the things that … everybody rather prepares them for load shedding, but one of the things that is affected by load shedding is your networks. So, the networks are off when there’s load shedding.’ (Participant 7, female, 9 years’ experience)

Another significant barrier was the overburdening of pharmacy services. The introduction of new policies, particularly those involving medication changes, placed additional pressure on pharmacy staff, leading to stock shortages and longer patient waiting times. Some facilities were further disadvantaged by the complete absence of on-site pharmacy services, which delayed patient care and limited medication access.

One participant shared:

‘Then you also need to think of your medication. When you look specifically at this facility, the implementation of ART [*antiretroviral therapy*] was not so easy. If I can talk specifically about our facility, because we don’t have a pharmacy at our facility … An ideal clinic has an extensive list of medications that they indicate that you must have; it’s like a must-have essential factor at the facility. So, then I saw that the ART [*antiretroviral therapy*] medication is included. You see, so that could not make me not to have that medication for my clients at the facility.’ (Participant 1, female, 11 years’ experience)

Finally, the timeframe allocated for policy implementation was flagged as problematic. In some cases, facilities were notified of a new policy mere days before its expected rollout, leaving inadequate time for training and preparation, which inevitably delayed the implementation process:

‘You often hear about a new policy only days before it’s meant to take effect. Sometimes, we’re invited to training on the 30th of March for a policy expected to go live on the 1st of April. It’s very difficult to meet such tight deadlines.’ (Participant 4, female, 17 years’ experience)

### Theme 4: Training

Training plays a pivotal role in the successful implementation of new health policies. If healthcare workers receive comprehensive, timely and appropriate training, the process of meeting policy indicators and targets can be significantly streamlined. Conversely, gaps in training, whether because of logistical issues or operational constraints, can delay or hinder the effective rollout of policies.

#### Sub-theme 4.1: Logistical barriers to training

Participants identified a variety of logistical obstacles that prevented staff from receiving necessary training ahead of policy implementation. While some participants reported that all professional nurses in their facilities were trained in Nurse-Initiated Management of Antiretroviral Therapy (NIMART), others highlighted the lack of consistent NIMART training coverage across staff.

Several factors were highlighted as barriers to efficient training: inconsistent availability of training opportunities, administrative holdups in processing training applications, and the absence of an independent registration system that would allow staff to self-enrol for training when needed. Participants expressed the view that allowing self-registration could reduce unnecessary delays and improve access to essential training:

‘If self-registration were possible, the process would be much simpler. Instead, you have to complete forms, wait for approval, and deal with multiple small restrictions. If training is essential at the primary healthcare level, why not make it more accessible?’ (Participant 8, female, 2 years’ experience)

Several participants found that insufficient or delayed training before policy rollout ultimately slows the implementation process at the facility level, as staff are left underprepared:

‘The problem is that we often don’t have enough time between learning about a new policy and having to implement it. The gap in preparation creates challenges at the facility, and delays tend to occur.’ (Participant 3, female, 10 years’ experience)

#### Sub-theme 4.2: Training attendance challenges

Participants discussed several challenges related to staff attendance at training sessions. Service demands and operational pressures often prevent facilities from releasing staff for training, as doing so may necessitate employing locum staff, which incurs financial costs. Previously, a skills development fund covered locum expenses, but with its discontinuation, facilities must now allocate these costs from their budgets, creating financial strain. The limited availability of online training that could serve as an alternative to in-person sessions was also observed. Even when online training was accessible, technical difficulties, such as malfunctioning links, reduced its effectiveness. Despite these challenges, most participants supported online training as it was less likely to disrupt services:

‘I prefer actually the online because they can then do it in their own time, and you can structure it versus the physical one, which is a long time out. That just presents a massive problem because then you have to cut your numbers here and then your patient complaints go up. So that really is a bit of a struggle.’ (Participant 8, female, 2 years’ experience)

However, some participants advocated for more innovative training approaches, such as webinars, automated e-learning modules, and virtual simulations. They emphasised the need for modern, technology-driven training methods that include automated assessments and structured evidence of completion:

‘I want you to do this, and then by this time you need to complete and provide me with the evidence that you’ve actually done the completion, but now it is this form and that form and what and send it to substructure. No innovative training in place.’ (Participant 8, female, 2 years’ experience)

## Discussion

This study explored the challenges experienced by HIV managers in implementing HIV policies at primary healthcare (PHC) facilities, and the findings contribute to a growing body of literature that emphasises how various systemic, organisational, and human factors influence the success of health policy implementation, especially in resource-constrained settings. The challenges identified in this study mirror those found in global literature, highlighting both commonalities and variations in the experiences of HIV managers across different regions.

The findings of this study should also be understood in relation to national HIV policy expectations in South Africa. Frameworks such as the National Strategic Plan for HIV, TB and STIs 2023–2028 (South African National AIDS Council 2023), the Integrated Clinical Services Management (ICDM) framework (National Department of Health, 2016b) and the National HIV Programme Guidelines (National Department of Health 2023) outline clear standards for HIV prevention, treatment continuity, chronic care integration and health system readiness. However, the challenges described by HIV managers in this study, particularly shortages in human resources, infrastructure limitations, inadequate training and inconsistent dissemination of policy updates, demonstrate gaps between these national expectations and the realities of implementation at the PHC level. Although the Department of Health’s Knowledge Hub provides online training and implementation tools intended to support policy uptake, participants highlighted limited access, inconsistent utilisation and digital literacy barriers. These findings indicate a need for stronger operational support and more structured, system-wide mechanisms to help PHC facilities align practice with national policy intentions.

One significant barrier revealed in this study is human resource challenges. Staff shortages, absenteeism, and an increasing patient load are among the key constraints HIV managers face in effectively implementing HIV policies. This finding resonates with the research of Plazy et al. (2017) in Côte d’Ivoire, where healthcare workers expressed concerns that rising patient numbers would compromise the quality of care, a fear that stems from being overburdened. A similar study by Mupara, Ehlers and Ehlers (2022) in Malawi also found that staff shortages, particularly in rural health centres, significantly undermined the successful application of HIV treatment guidelines. Inadequate human resource capacity has long been acknowledged as a central factor influencing health policy implementation. Khan and Khandaker (2016) argue that human resources are a primary input in the execution of health policies, and this is further supported by South Africa’s National Policy Development Framework (2020), which underscores the importance of adequate human resources for achieving health policy objectives. The findings of this study are consistent with those of Tshililo, Davhana-Maselesele and Maputle (2019) in Limpopo, South Africa, where staff shortages were found to hinder comprehensive HIV care and early detection of opportunistic infections. On a broader scale, a multicountry review by Cometto et al. (2018) highlighted that an overstretched workforce often causes fragmented service delivery across sub-Saharan Africa. In Canada, Bryant-Lukosius et al. (2015) reinforced the importance of adequate staff-to-patient ratios in ensuring safe, efficient and evidence-based care in PHC settings, which underscores the global dependence on robust human resource capacity for successful policy implementation.

The study also identified that staff knowledge and attitudes play a crucial role in the success or failure of HIV policy implementation. Healthcare workers who lack sufficient knowledge or confidence often display fear, hesitation and resistance when confronted with new policies. This finding aligns with the WHO (2018) scoping review, which concluded that a lack of knowledge and confidence, especially in under-resourced and rural facilities, poses significant barriers to the implementation of new health interventions. In South Africa, Muthelo, Maluleke and Lebese (2021) found that negative attitudes among professional nurses, coupled with inadequate training, were substantial obstacles to effective policy implementation. Studies in Rwanda and Kenya have similarly highlighted the link between knowledge gaps, attitudes and policy adherence. Osei and Lee (2018) found that resistance to change often stems from limited engagement and inadequate training, which ultimately impacts the sustainability of policy-driven programmes. Furthermore, Cancedda et al. (2015) in Rwanda emphasised the importance of continuous education and knowledge-sharing in empowering healthcare workers to implement policies effectively. Makhado et al. (2020) also highlighted that policy implementation thrives in environments where staff members are motivated, trained, and included in the policy change process, rather than being passive recipients of top-down directives. The findings of this study reinforce the global understanding that investing in the development of healthcare workers’ knowledge and attitudes is critical to improving policy uptake and long-term success.

Infrastructure and equipment shortages were another major challenge faced by HIV managers, and these issues were consistently raised throughout the study. Inadequate consultation rooms, poor ventilation, incompatible IT equipment and persistent issues with health information systems (HIS) were among the recurrent barriers to policy implementation. These findings are consistent with the Management Model proposed by Khan and Khandaker (2016), which emphasises that infrastructure and technology are vital components for the successful execution of health policies. A study by Crowley and Stellenberg (2014) in KwaZulu-Natal, South Africa, similarly found that logistical, spatial, and infrastructural limitations hindered the ability of PHC facilities to offer integrated HIV services. In addition, the Tanzanian experience, as reported by Shabani et al. (2017), demonstrated how infrastructure and equipment limitations hampered the effective implementation of the Option B + PMTCT policy, directly affecting maternal and infant outcomes. Operational challenges such as load shedding, identified in this study, also mirrored issues faced by health facilities in Zimbabwe and Zambia, where power outages disrupted patient record management, laboratory services and medication dispensing (Moyo, Madziyire & Mapanga 2020). In response to such challenges, the World Health Organization (2016) has recommended that health systems be designed to be climate- and energy-resilient, underscoring the importance of robust infrastructure for sustaining healthcare delivery under varying conditions. Moreover, pharmaceutical service delivery was also compromised in this study, particularly when pharmacists were absent, and ART dispensing had to be delegated to unqualified or inadequately trained staff. This finding is consistent with research conducted in the Eastern Cape by Bobbins, Burton and Forgarty (2020), who identified similar task-shifting trends in pharmaceutical services because of pharmacist shortages, raising concerns about the long-term safety and sustainability of HIV treatment programmes.

Training challenges emerged as a significant concern in the study, with logistical barriers to organising and attending timely training courses impeding staff readiness for policy rollouts. This finding aligns with the broader challenges identified by Ahmat et al. (2022), who highlighted resource constraints in health systems across 12 African countries, including Chad, impacting service delivery and workforce capacity.

The study also found that technology-enabled training, such as simulations and online modules, could mitigate logistical barriers, but challenges such as computer illiteracy and poor internet connectivity posed additional obstacles to e-learning (Baniasadi, Ayyoubzadeh & Mohammadzadeh 2019; Zhang et al. 2018). In Canada, Morton et al. (2021) emphasised the potential of technology-enhanced training to reduce implicit biases and improve clinical competencies, although they noted the need to tailor these platforms to the capabilities of users. This study’s participants highlighted the need for flexible, accessible, and ongoing training, aligning with global recommendations for blended learning models that combine in-person and digital education (WHO 2018). In addition, the withdrawal of the skills development fund in South Africa, which had previously supported staff replacements for training attendance, exacerbated this challenge. The need for sustainable funding mechanisms to support training as a core component of health system strengthening has also been highlighted by Cometto et al. (2018).

Overall, the findings of this study resonate with global literature, underscoring the multifaceted nature of policy implementation challenges. Human resource shortages, insufficient training, infrastructural constraints, negative staff attitudes, and operational inefficiencies such as poor health information systems are not unique to South Africa, but are common obstacles faced in various global contexts. The success of HIV policy implementation is dependent not only on the quality and content of the policy itself, but also on the enabling environment, which includes human resources, infrastructure, attitudes, and ongoing professional development. Insights from Africa, Asia and North America confirm that these challenges are deeply interconnected and require a coordinated, system-wide response to achieve meaningful and sustainable improvements in HIV policy implementation at the PHC level.

## Strengths, limitations and areas for further research

A key strength of this study lies in its commitment to maintaining rigour and trustworthiness despite several logistical and environmental challenges during data collection. Although some managers cancelled scheduled interviews multiple times, the researcher maintained consistent follow-up, which extended the data collection period by an additional month. Despite this delay, the quality, depth, and consistency of the data remained uncompromised.

A key strength of this qualitative exploratory–descriptive study is the depth and richness of the data, which enabled a nuanced understanding of HIV policy implementation within PHC settings. Despite logistical challenges, such as interview cancellations, environmental noise at some facilities, and an extended data collection period, the researcher maintained rigour through persistent follow-up, careful scheduling, and pausing interviews when necessary to preserve privacy and data quality.

However, several limitations must be acknowledged. As with most qualitative studies, the findings of this study are context-bound and shaped by participant perspectives, which may limit their transferability. The gender imbalance within the sample, reflecting the workforce composition rather than sampling bias, restricted the representation of male viewpoints. Additionally, the study was designed as an inductive enquiry and did not adopt the Consolidated Framework for Implementation Research (CFIR) as a guiding analytical model. While appropriate for exploring operational-level experiences, this limits the study’s ability to systematically analyse multilevel implementation determinants. Future research could benefit from applying CFIR or similar frameworks to broaden system-level insight across policy, organisational and individual domains.

## Recommendations

Firstly, structured mentorship and continuous supportive supervision should complement formal training to build clinical competence and strengthen teamwork. Such an approach encourages reflective practice and enables healthcare workers to develop trust and resilience when adapting to new policies. Ploeg et al. (2019) highlight that mentorship enhances professional confidence and facilitates the integration of evidence-based practices in day-to-day care delivery.

Secondly, it is recommended that all professional nurses receive training in NIMART. This would not only enhance workforce flexibility, particularly during staff rotations or shortages, but also ensure continuity of ART services across PHC settings. Tshililo et al. (2019) emphasise that embedding NIMART within the broader chronic disease management model can reduce HIV-related stigma and support integrated service delivery.

Thirdly, improving digital literacy is essential to enhance the adoption and utilisation of electronic health information systems. Providing basic information and communication technology (ICT) training to all staff will support efficient service delivery and contribute to meeting the standards outlined in the Department of Health’s Ideal Clinic framework (Department of Health 2018).

Furthermore, integrating HIV services into general PHC streams rather than maintaining them as standalone services could promote continuity of care, reduce stigma, and strengthen the holistic management of patients.

Lastly, the use of telehealth for stable HIV patients offers promising potential to ease the workload at PHC facilities while enhancing patient access to care. Studies by Monaghesh and Hajizadeh (2020) demonstrate that telehealth interventions can maintain service quality while improving convenience and system resilience, particularly in under-resourced environments.

Adopting these strategies would help to address both human and systemic barriers to health policy implementation and create a more enabling environment for delivering comprehensive HIV services at the primary healthcare level.

## Conclusion

Taken together, the findings of this study underscore the multifaceted and interconnected nature of the barriers to HIV policy implementation at the PHC level. Human resource shortages, insufficient training, inadequate infrastructure, and poor health information systems all contribute to the challenges experienced by HIV managers in implementing HIV policies. Addressing these barriers requires a comprehensive, multipronged approach that includes investment in human resources, the development of efficient training systems, and the strengthening of health infrastructure and information systems. By tackling these structural and operational challenges, it may be possible to improve the quality of HIV care and enhance the effectiveness of HIV policy implementation at PHC facilities, ultimately contributing to better health outcomes for people living with HIV.
